# Which of the Physiological vs. Critical Speed Is a Determinant of Modern Pentathlon 200 m Front Crawl Swimming Performance: The Influence of Protocol and Ergometer vs. Swimming Pool Conditions

**DOI:** 10.3390/sports10120201

**Published:** 2022-12-06

**Authors:** Sabrina Demarie, Emanuele Chirico, Veronique Billat

**Affiliations:** 1 Department of Movement, Human and Health Sciences, University of Rome “Foro Italico”, 00135 Rome, Italy; 2 Department of Sciences et Techniques des Activités Physiques et Sportives (STAPS), University of Paris-Saclay, 91042 Evry, France

**Keywords:** health protection, swimming flume, lactate threshold, energy cost, training monitoring, performance analysis, swimming performance, VO_2_max, critical velocity, multisport, training programs

## Abstract

Background: Modern pentathlon includes horse riding, fencing, swimming, shooting and cross-country running. Events can last many hours during which the athletes face almost maximal energy and physiological demands, and fatigue. Early recognition and prevention of injuries and overuse syndromes can be achieved by refining the individual training loads. The purpose of the study was to determine which parameter could be the most accurate predictor of swimming working capacity determinants in pentathletes. Methods: Fourteen male pentathletes performed a continuous maximal incremental test in the swimming flume ergometer to measure peak oxygen uptake (VO_2_peak), and five swimming tests in a 50 m swimming pool to detect critical velocity (CV); velocity at 2 and 4 mM·L^−1^ of blood lactate (v2, v4) and energy cost (EC). Results: The 200 m swimming time was 2:18–2:32 m:s (340 FINA points). CV was 1.21 ± 0.04 m·s^−1^, v2 was 1.14 ± 0.09 and v4 1.23 ± 0.08 m·s^−1^. VO_2_peak was 3540.1 ± 306.2 mL·min^−1^ or 48.8 ± 4.6 mL·kg^−1^·min^−1^. EC at 1.24 m·s^−1^ was 45.7 ± 2.4 mL·kg^−1^·min^−1^. Our main finding was the large correlation of CV with 200 m swimming performance; Conclusions: Among all the protocols analysed, CV is the most predictive and discriminative of individual swimming performance in this group of pentathletes. It appears as the most suitable test to constantly refine their swimming training loads for both performance enhancement and health promotion.

## 1. Introduction

In multiple sports, such as modern pentathlon, events can run up to many hours, which means that the athletes face energy and physiological demands almost at their maximum, and can accumulate a high load of fatigue [1,2]. The high training loads and high levels of training and competition stress endured by many athletes make it important to manage the risks associated with the possible negative outcomes, while maintaining optimal physiological and psychological health [3,4,5,6]. Training and competition stress can result in temporary decrements in health status, derived from increased muscle damage, impairment of the immune system, imbalances in anabolic–catabolic homeostasis, alteration in mood and reduction in neuromuscular function [7,8]. In order to reduce non-functional overreaching, illness, and injury risks and to guide the training process effectively, coaches must have a systematic knowledge of how individuals respond to given sets of training loads [9]. Performance tests replicating the athlete’s competition would be the more predictive and discriminative of individual stress placed on the athletes [10,11,12].

In competition order, events include horseback riding (show jumping), fencing (épée), swimming (200 m) and combined shooting and cross-country running (laser-run). The contribution to the final score is 15% for fencing, 20% for both equitation and swimming and 45% for the laser-run. Laser-run is the last phase that is faced in pentathlon competition and is considered the most important for the outcome of the overall event [13,14,15,16,17]. Even though the swimming phase accounts for 20% of the overall score only, it is always the last discipline competed before the laser-run. Since the effort exerted during previous events can influence the outcome of the following ones, achieving higher efficiency, while accumulating less fatigue, in swimming, can be profitable to both the swimming phase score and the laser-run performance [18,19,20,21].

Due to the multiple energetic pathways involved in the 200 m front crawl, researchers, coaches, and athletes face challenges in analysing and training for it. Many velocity thresholds and physiological indices are valid and reliable predictors of swimming performance and are commonly used for the evaluation and the adjustment of the training pace [22,23].

Peak oxygen uptake and energy cost assessments were proved to be good predictors of swimming performance [24,25]. A positive relationship between VO_2_peak and 200 m front crawl swimming speed has been observed and peak oxygen uptake has been proposed as a good predictor of the 200 m swimming performance [26]. The aerobic energy cost of swimming can be estimated measuring the oxygen uptake at the intensity performed at a steady state at intensities below the lactate threshold [27,28,29]. When lactate accumulation is detected, the contribution of the anaerobic system can be calculated and expressed in ml·min^−1^·kg^−1^ [30,31]. However, the direct in-water measurement of oxygen uptake requires swimmers to breathe through a snorkel, which hinders diving starts and underwater gliding, possibly impairing the ecological validity of the measurements [27,30,32,33,34].

The onset of blood lactate accumulation, defined as the intensity of exercise at which blood lactate reaches 4 mM·L^−1^ during an incremental exercise test, has been widely used for training prescription and monitoring [35,36]. The velocity corresponding to the blood lactate concentration of 4 mmol·L^−1^ was proven to determine 59% of the variance in the 200 m front crawl performance of expert male swimmers [23]. However, to evaluate anaerobic behaviour and training intensities, it has been suggested that individual assessments, rather than exercise intensities at fixed blood lactate levels, would be more effective [25]. It was shown that swimming at critical speed corresponds to the velocity at the maximal lactate steady state and that the plasma lactate concentration values greatly varied depending on the swimmer’s distance of competition. These results add a great deal of support to the position that, because of the wide inter-individual variability in lactate responses to a comparable exercise intensity, the use of fixed lactate values as threshold criteria could be inappropriate [37]. Indeed, training-induced improvements in lactate parameters were associated with improvements in maximal 200 m swimming test times during training sessions, but no direct correlation between blood lactate profiling and international competition performance were found [38].

Most of the above-mentioned methodologies require cumbersome and costly equipment, are time consuming and disruptive of the training routine [39]. At each training session, pentathletes train multiple disciplines with short recovery periods between them, and often they do not have the same technical support as élite swimmers [2,40]. The need for costless, not time-consuming and ecological tests may have precluded the wider implementation of the critical velocity concept in swimming applied practice [41]. Anaerobic critical velocity has been reported to be an important indicator of performance in the 200 m swimming events [42]. For most swimmers, critical speed can be obtained from two criteria efforts only, by the measurement of the swimming best times on distances from 50 to 800 m. It could be used as relevant criteria to predict performances and evaluate physiological and technical status and to monitor and give advice concerning training [35,43,44,45,46,47,48]. However, the critical swimming speed does not appear to represent the maximal swimming speed that can be maintained over a long period of time without a continuous rise in blood lactate concentration and is hardly tolerable according to the rate of perceived exertion [49].

The purpose of the present study was to determine which parameter could be considered the most accurate predictor of swimming performance in the pentathlon competition. To provide a deeper understanding of pentathletes’ physiological thresholds and energy cost several swimming test protocols were administered in a swimming flume ergometer and in ecological conditions in a 50 m swimming pool. Some tests required the use of respiratory gas and/or blood lactate analysers, while the critical velocity assessment tests were administered in ecological conditions of free swimming in the pool. The specific aim of this study was to validate the most practical protocol allowing the most significant index of performance to be obtained to help trainers who do not have access to a flume nor the time to achieve long or maximal tests.

## 2. Materials and Methods

Fourteen male pentathletes (age 19.4 ± 0.9 years; height 182.1 ± 4.0 cm; weight 73.8 ± 7.2 kg) competing and training at Tier 4 Elite/International Level [50], underwent several swimming testing sessions. The inclusion criteria were having competed at international level in the last year, and medical eligibility for competitive pentathlon. The exclusion criteria were interruption of training for more than two consecutive weeks in the previous three months; regular intake of medications and/or the presence of chronic pathologies. The participants provided informed written consent before data collection. The experimental protocol was performed according to the Declaration of Helsinki and was approved by the local Ethics Committee (CAR 129/2022, 8 June 2022).

### 2.1. Procedures

Athletes underwent several swimming tests, either in a 50 m indoor swimming pool or in a swimming flume. In both conditions, the water temperature was kept at 27 °C. All sessions were held over 4 consecutive days, at the same time of the day, at least 4 h after having eaten, in fully rested and hydrated conditions. Athletes performed a standard 30 min warm-up, 10 min dry land stretching and 20 min slow self-paced front crawl before each session. Each swimming test undergone in the same day was separated by full active recovery, determined by the return of heart rate individually and manually assessed to rest level. Before each test, all participants rested seated outside the water for 5 min.

The athletes’ best time of the competition’s swimming phase was communicated by their coaches, as approved by the Italian Modern Pentathlon Federation.

When blood lactate was measured, arterialized blood capillary samples (20 µL) were taken at the earlobe. After warm-up, blood samples were collected right before the start, at the end and 3, 6 and 9 min after each test. Lactate concentration was measured using an amperometric analyser (EBIO Plus; Eppendorf, Hamburg, Germany). The difference between the peak and basal values was used to calculate the accumulation of blood lactate of each test.

When oxygen uptake was measured, it was continuously assessed breath by breath for 5 min before the start and throughout the tests. Gas exchanges were measured through a snorkel with a low airflow resistance (Dalacqua; Cosmed, Rome, Italy) connected to a portable gas exchange analyser (K4 b^2^; Cosmed, Rome, Italy) that was suspended over the water (at a 2 m height) by a steel cable minimizing disturbances of the normal swimming movements. In the flume, the cable supporting the metabolimeter was fixed at the ceiling, while in the pool, it was carried alongside the swimming athlete by trained personnel. To reduce inter-breath fluctuations, the VO_2_ data were averaged every 5 s for further analysis. The gas exchange equipment was calibrated before each test following standardized procedures [51,52].

#### 2.1.1. Tests in the 50 m Pool with a Stopwatch Only

##### The 100, 200, 300 m and Critical Velocity (CV)

The best times of the maximal 100 m (test 1.1), 200 m (test 1.2) and 300 m (test 2.2) front crawl swimming were utilised to determine the subject’s CV. From the three timed distances, the linear relationship between time and distance were drawn, and the CV was determined as the slope of the relationships’ best fit line. Maximal speed was attained according to athletes’ best individual performance and own experiences, whilst coaches encouraged them to swim at their best effort. All tests comprised free swimming with standard starts and turns and were manually timed.

#### 2.1.2. Tests in the 50 m Pool with a Stopwatch and Blood Lactate Measurements

##### Maximal and 90% 200 m and Velocities Corresponding to the 2 and 4 mM·L^−1^ of Lactate Accumulation (v2 and v4)

Subjects underwent a maximal 200 m front crawl tests. After complete recovery, they were instructed to swim for 200 m at a constant submaximal velocity corresponding to 90% of their 200 m maximal velocity. The test was paced by acoustic feedback (every 25th m) and each athlete’s coach provided continuous visual feedback. To represent the athlete’s individual lactate/velocity profile, from the results of the maximal and submaximal 200 m swimming, lactate accumulation values were interpolated with the mean velocities maintained during the tests. The individual equation of the linear relationship among lactate accumulation and mean swimming velocities was used to determine the velocities corresponding to the accumulation of 2 and 4 mM·L^−1^ of lactate (v2 and v4).

#### 2.1.3. Tests in the Flume with Blood Lactate and VO_2_ Measurements

##### Energy Cost at Submaximal Velocity (EC)

Oxygen uptake was continuously measured during 6 min front crawl swimming at a constant submaximal velocity corresponding to 50% of the difference between vVO_2_peak, and the critical velocity (CV). The mean oxygen uptake (EC) measured in the last 2 min of the 6 min constant velocity front-crawl swimming test in the flume at v50% (session 4) was considered the gross aerobic energy cost of submaximal swimming above the CV. The net aerobic EC (ECnet) was calculated subtracting the basal oxygen uptake, measured in the last 2 min of rest before starting the test (ECrest), to the EC. The contribution of the anaerobic system (ECLanet) was calculated as the lactate accumulation (∆La in mM·L^−1^) multiplied by the energy equivalent of lactate (LaEq in mlO_2_ mM^−1^·kg^−1^) and divided by the exercise duration (ExTime in min); the metabolic power output derived from anaerobic lactic metabolism is, thus, also expressed in ml·min^−1^·kg^−1^ [30,31]. The net total energy cost (TotECnet) of front crawl swimming in the flume at v50% was calculated by adding ECLanet to ECnet. The percentage contribution of ECLanet to TotECnet was also calculated. All ECs were analysed in relative to body weight values.

##### Peak Oxygen Uptake (VO_2_peak)

To measure peak oxygen uptake (VO_2_peak) and the corresponding velocity (vVO_2_peak) a continuous multistage maximal incremental test was carried out in a swimming flume. Starting from 65% of individual maximal 200 m swimming velocity, the velocity was increased by 0.05 m·s^−1^ every minute until exhaustion (i.e., when the subjects were unable to swim at the required velocity). Since swimming at increasing velocities in the flumes represents a challenge even for high-level swimmers, there was no expectation that pentathletes would endure long enough to attain the steady state of oxygen uptake that would have been necessary to establish their VO_2_max. Therefore, peak oxygen uptake was considered the maximal value reached at the end of the test, given that the following criteria were met: exhaustion of the subject; heart rate ± 5 beats per minute with respect to the maximal theoretical heart rate; respiratory exchange ratio above 1.1; peak lactate accumulation above 8 mM·L^−1^ [53,54]. Test stages’ timing was recorded by the metabolimeter acquisition system. The velocity of the last stage endured at least 30 s was considered as vVO_2_peak.

### 2.2. Statistical Analysis

All values are reported in the text as the mean ± SD. The statistical analyses were performed using the Statistical software SPSS (version 26.0; SPSSTM Inc., Chicago, IL, USA). Data were screened for normality of distribution and homogeneity of variance using a Shapiro–Wilk test for Normality. To analyse the difference between variables measured either Repeated-measures ANOVA or a Dependent pair *t*-test for the paired sample were used, while a Wilcoxon Signed Ranks Test was used when the variables were not normally distributed. A Bonferroni post-hoc test was used when a significant main effect for velocity was observed. In the first analysis, a dependent pair *t*-test was used to analyse the difference between oxygen uptakes obtained in the swimming pool and the same measures obtained in the flume (*p* < 0.05). The Wilcoxon Signed Ranks Test was applied to investigate on the difference between the lactate accumulation in the swimming pool and the flume tests (*p* < 0.05). Thirdly, a Repeated Measured ANOVA was used to analyse the difference in velocity values of the whole test sessions (*p* < 0.05). The effect size was calculated using the partial eta squared (η^2^) in the repeated-measures ANOVA and Cohen’s d in dependent pair *t*-test.

To explore the relationship between competition swimming performance and the other variables a one-dimensional linear regression model was performed. Significance level for all analyses was set at *p* = 0.05.

## 3. Results

### 3.1. Normality and Effect Size

All results were normally distributed, except for lactate accumulation values. Therefore, each statistical procedure was conducted as depicted in Table 1.

### 3.2. Best Time of the Competition’s Swimming Phase

The best times achieved by our subjects in the 200 m front-crawl swimming phase of a pentathlon competition closest to the test dates were 2:26 ± 0.04 min·s^−1^. Their 200 m swimming best time ranged from 2:18 to 2:32 m:s, corresponding to 340 FINA points (https://www.fina.org/swimming/points; accessed on 15 June 2022.). They performed a swimming competition phase 18% slower than the co-national and 21% slower than All Nations’ participants to the previous year’s World Championship.

#### 3.2.1. Tests in the 50 m Pool with a Stopwatch Only

##### The 100, 200, 300 m and Critical Velocity (CV)

The best front crawl swimming times of the 100 m (test 1.1), 200 m (test 1.2) and 300 m (test 2.2) resulted in 1.6 ± 0.1, 1.5 ± 0.1 and 1.3 ± 0.04 m·s^−1^, respectively. From these velocities, a mean critical velocity for the whole group of 1.21 ± 0.04 m·s^−1^ was calculated.

#### 3.2.2. Tests in the 50 m Pool with a Stopwatch and Blood Lactate Measurements

##### Maximal and 90% 200 m and Velocities Corresponding to the 2 and 4 mM·L^−1^ of Lactate Accumulation (v2 and v4)

According to the 200 m swimming at maximal velocity (1.5 ± 0.1 m·s^−1^) and 200 m swimming at 90% of maximal velocity (1.4 ± 0.05 m·s^−1^) tests’ results, each subject’s lactate/velocity profile was created. From the individual profile, the velocities corresponding to the lactate accumulation of 2 mM·L^−1^ and 4 mM·L^−1^ were calculated and the mean results for the whole group were 1.14 ± 0.09 and 1.23 ± 0.08 m·s^−1^, respectively.

#### 3.2.3. Tests in the Flume with Blood Lactate and VO_2_ Measurements

##### Energy Cost at Submaximal Velocity (EC)

The EC measured as mean oxygen uptake of the last 2 min of the 6 min constant velocity front crawl swimming in the flume at v50% (1.24 ± 0.04 m·s^−1^) was 45.7 ± 2.4 mL·kg^−1^·min^−1^, a value significantly lower than VO_2_peak (t = 2.379; *p* = 0.03). ECrest, measured in the last 2 min of rest before starting the constant submaximal velocity test, was 5.1 ± 1.2 mL·kg^−1^·min^−1^. The ECnet calculated subtracting ECrest from EC was 40.7 ± 3.1 mL·kg^−1^·min^−1^.

The lactate accumulation (3.9 ± 1.0 mM·L^−1^) was significantly lower than the Lapeak measured in the incremental test in the flume (z = −3.296; *p* = 0.001). The contribution of the anaerobic system (ECLanet), calculated as the lactate accumulation (∆La = 3.9 ± 1.0 mM·L^−1^) multiplied by the energy equivalent of lactate (LaEq = 2.7 mlO_2_ mM^−1^·kg^−1^) and divided by the exercise duration (ExTime = 6 min) was 1.8 ± 0.4 mL·kg^−1^·min^−1^. The net total energy cost (TotECnet) of front crawl swimming in the flume at v50% was calculated by adding ECLanet to ECnet and resulted in 42.4 ± 3.3 mL·kg^−1^·min^−1^ in absolute values and 97.9 ± 12.4 % of the netVO_2_peak. The percentage contribution of ECLanet to TotECnet resulted in 4.1 ± 0.9%. All ECs were analysed relative to body weight values.

##### Peak Oxygen Uptake (VO_2_peak)

The test starting velocity (vStart) was calculated for each subject corresponding to 65% of individual maximal 200 m swimming velocity; the mean starting velocity was 0.97 ± 0.07 m·s^−1^. The peak velocity (vPeak) reached was 1.27 ± 0.07 m·s^−1^ and the mean test duration (Tlim) resulted in 6:28 ± 0.1 min·s. The peak lactate (Lapeak) accumulation was 8.7 ± 0.6 mM·L^−1^ and the VO_2_peak reached was 3540.1 ± 306.2 mL·min^−1^ or 48.8 ± 4.6 mL·kg^−1^·min^−1^. The netVO_2_peak assessed as the difference between VO_2_peak and the mean value measured in the last 2 min of rest before starting the incremental test was 43.8 ± 5.0 mL·kg^−1^·min^−1^.

### 3.3. Differences among the Swimming Velocities

**Figure 1:**
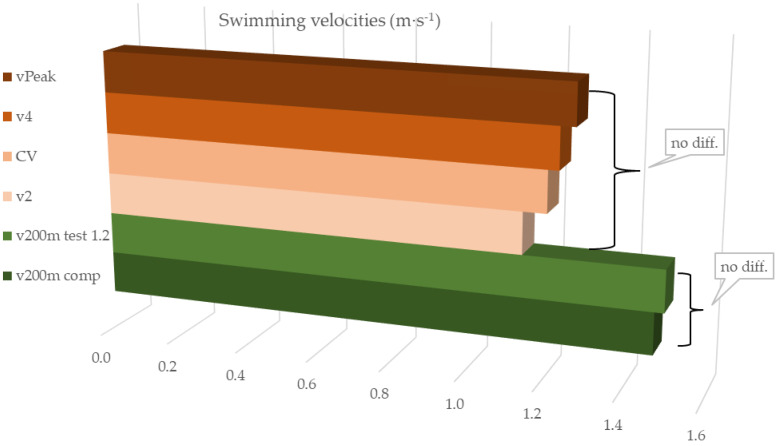
*Competitive and tests swimming velocities.* vPeak: highest velocity of test 3; v2, v4: velocities corresponding to the lactate accumulation of 2 mM·L^−1^ and 4 mM·L^−1^; CV: critical velocity; v200m test 1.2: swimming velocity of 1.2; v200m comp: 200 m swimming velocity in competition. No diff.: *p* > 0.05.

Swimming velocities showed statistically significant different values (F = 333.095; *p* < 0.001). However, post hoc analysis revealed that CV was not significantly different from v2, v4 and vVO_2_peak. Additionally, the velocities measured in the swimming competitions (v200m competition) and the velocities reached during the 200 m maximal front crawl test (v200m max) were not significantly different between them, ensuring that athletes achieved their best swimming times in test 1.2. Non-significant differences are depicted in Figure 1 with the same filling pattern and the arrows’ connections.

### 3.4. Correlation of Measured Variables with Performance

**Figure 2:**
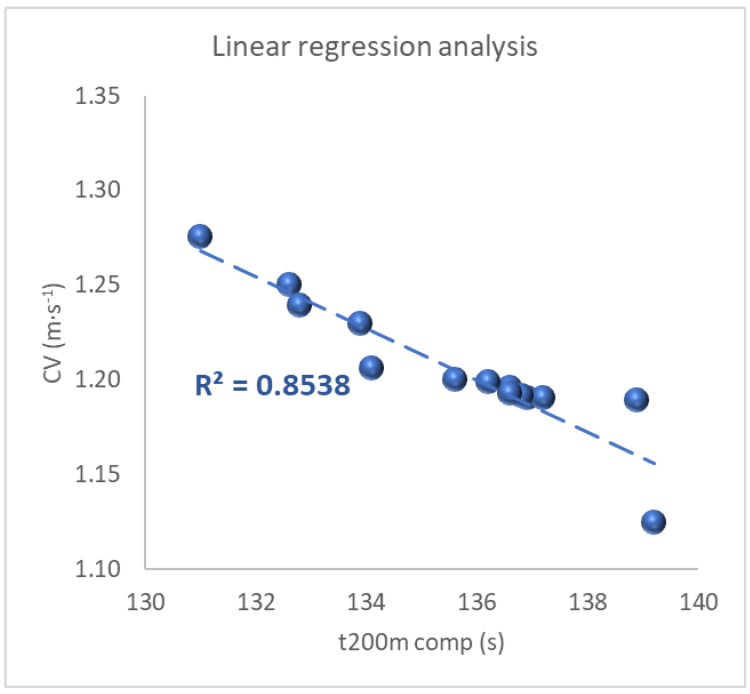
Regression analysis among the performance of the pentathlon competition swimming phase and CV. CV: critical velocity; t200m comp: 200 m swimming time in competition.

The performance time of the 200 m pentathlon competition swimming phase displayed a strong inverse relationship with CV (r = −0.920; *p* < 0.001) as represented in Table 2 and in Figure 2. CV accounted for 85% of the variability in performance.

## 4. Discussion

The aim of the present study was to test different maximal and submaximal swimming evaluation protocols in elite pentathletes, for the purpose of estimating which can more accurately predict pentathletes’ swimming competition performance. Although the pentathletes included in the study competed at Elite/International level, swimming was not the most important phase of their competitions [50]. Their 200 m swimming best-time corresponded to a low swimmer’s word ranking of 340 FINA points. They performed a swimming competition phase 18% slower than their co-national and 21% slower than All Nations’ participants to the previous year’s World Championship.

### 4.1. Tests in the 50 m Pool with a Stopwatch Only

#### Critical Velocity (CV)

Our main finding was the large inverse relationship that CV showed with the 200 m front-crawl swimming competition times (Figure 2). Athletes with the higher CV tended to have lower 200 m performance times. Accordingly, it has been proposed that 200 m performances can be partly explained by the level of aerobic fitness indicated by the CV [55]. It was shown that in well-trained swimmers the CV corresponded to the velocity at the maximal lactate steady state (vMLaSS), while a slightly increased intensity (approximately 0.02 m∙s^−1^) was sufficient for plasma lactate to accumulate throughout the swimming sets of either 6 × 400 m or 12 × 200 m. Critical speed has been proposed to represent an intensity close to the upper limit of oxidative capacity representing a swimming velocity that is 80–85% of maximum 100 m velocity, or 90–95% of maximum 400 m velocity. CV is accepted as the theoretic maximal swimming speed that can be maintained without exhaustion for a long period of time and it was expected to correspond to v4. In the present study, CV resulted in being placed in between v2 and v4 and no significant differences were found among the three velocities. Indeed, the evaluation of the critical velocity has been proposed as valuable in predicting the best possible race times for a given distance and for choosing race tactics that should optimize the performance outcome [56]. In agreement, in the current study, some evaluation protocols were used to determine the most accurate predictor of pentathletes’ swimming performances and CV resulted in being the only parameter correlated with swimming times in competition.

CV can also be calculated within a single exercise bout by the 3-min all-out test (3MT) firstly developed for cycling by Burnley et al. [57], and then applied to swimming by Piatrikova et al. [41]. According to the latter study, the CV derived from the 3MT is comparable to the CV derived from conventional models. As a result, the 3MT can be regarded as a valid and reliable alternative protocol to estimate CV in highly trained swimmers and could represent a potential for the more widespread use of the CS concept.

### 4.2. Tests in the 50 m Pool with a Stopwatch and Blood Lactate Measurements

#### Maximal and 90% 200 m and Velocities Corresponding to the 2 and 4 mM·L^−1^ of Lactate Accumulation (v2 and v4)

In the present study, neither v2 nor v4 were correlated to the 200 m pentathletes’ swimming performance. It has been reported that, in front crawl swimmers, the individual anaerobic threshold corresponds to a blood lactate concentration of 3.5 mM·L^−1^, suggesting that the 4 mM·L^−1^ of blood lactate concentration adopted as a standard threshold does not always correspond to the ‘‘true’’ anaerobic threshold of the athlete examined [48]. The values of plasma lactate concentration at vMLaSS varies from 3.26 mM·L^−1^ for long- and middle-distance swimmers, to 11.5 mM·L^−1^ for sprinters. The wide range of plasma lactate elicited at vMLaSS, and the fact that many of the swimmers achieved plasma lactate values outside the 4 mM·L^−1^ lactate value, strongly implies that fixed blood-lactate values seem to be inappropriate for prescribing training intensities [37,58]. The fixed lactate 4 mM·L^−1^ value does not consider inter-individual differences, overestimating the real aerobic capacity of aerobically trained athletes [25]. In addition, the logic of the 4 mM·L^−1^ threshold rationale is limited for many reasons, one of them is that the relevant lactate concentration is that in the muscles and not in the blood stream [59]. Moreover, multiple linear regression models showed that v4 explained 59% of the 200 m front-crawl performance variance at the winter training season’s peak. This was supposedly due to the higher percentage of workout focused on the aerobic capacity in that specific period of the training season [23].

A correlation between lactate concentration and the intensity of each of the segments of a triathlon competition has been reported. The authors suggested that since swimming is the first modality of a triathlon, its lactate concentration levels can contribute to the accumulation of lactate on the subsequent competition modalities [60]. Therefore, even though the fixed lactate thresholds do not seem to be indicative of pentathletes’ performance in swimming, training to improve their anaerobic capacity could be beneficial to improve their resistance to long training and competition sessions.

### 4.3. Tests in the Flume with Blood Lactate and VO_2_ Measurements

#### 4.3.1. Energy Cost at Submaximal Velocity (EC)

The net EC, measured by subtracting the basal VO_2_ from the oxygen uptake of the last 2 min of the 6 min constant velocity front crawl swimming in the flume at 1.24 ± 0.04 m·s^−1^, resulted in 40.7 ± 3.1 mL·kg^−1^·min^−1^, a value significantly lower than VO_2_peak (t = 2.379; *p* = 0.03) and corresponding to its 94%. When the anaerobic contribution was accounted for, the swimmers’ energy cost increased by 4% and the total net energy cost rose to 42.4 ± 3.3 mL·kg^−1^·min^−1^. In our study, no correlations were found between swimming O_2_ cost and 200 m front crawl performance. In contrast, world-class male swimmers’ submaximal VO_2_ was correlated with their best 400 m competitive performance time (r = 0.67).

In studies concerned with the economy of competitive swimming the role of the anaerobic system to the total energy expenditure is not always considered. Therefore, the study of the energy expenditure based exclusively on the oxygen consumption might both underestimate the values and reduce the validity and utility of the measurements [31].

Differences between studies can also lie in the swimming level (pentathletes vs. swimmers) and in the race distances (200 m vs. 400 m). During a 200 m front-crawl swimming race, aerobic sources provide 58%, whereas in 400 m races oxygen sources provide 73% of the energy [61]. When energy expenditure was measured at a velocity of 1.2 m·s^−1^ for front crawl swimming, the mean VO_2_ values ranged from 25 mL·kg^−1^·min^−1^ for the most economical to 40 mL·kg^−1^·min^−1^ for the least economical swimmers [32]. In swimming, energy expenditure is largely determined by the athlete’s technique and biomechanical characteristics [1,62,63,64]. At a given speed, swimmers’ skill levels can make a substantial difference in energy expenditures; more proficient swimmers can expend 50% less energy than less proficient ones and 25% less than intermediates [29]. The energy cost of 400 m front crawl swimming was reported to remain constant throughout the 30 min test time duration at each of the three studied swimming intensities, below, at and above the MLSS. Despite the unchanged energy cost during test duration, at the above MLSS exercise intensity, the swimmers did not sustain their stroke length compared to the below and at MLSS intensities. They had to increase their stroke rate to maintain the required paced velocity, suggesting the importance of the ability to maintain biomechanical efficiency at every exercise intensity [65]. Using the manipulation of the stroke rate and stroke length might be one of the factors through which energy cost can be altered for a given velocity [27]. There is further evidence that high-training volumes and an extraordinary high-aerobic capacity do not seem necessary prerequisites for maximal performance in competitions lasting between 20 s and 5 min (50 m to 400 m events). Competitive swimming depends more on technical skills, such as power transfer to propulsion, than on other disciplines, such as running or cycling. Hence, a reduction in total training volume to focus on other performance-determining factors might contribute to a more economical training strategy [62].

Even though, for this group of pentathletes, the energy cost of swimming does not seem to represent a determinant of performance, an improvement in their swimming economy could be supposed to be beneficial. A lower oxidative metabolic rate for a given submaximal speed would represent a lower fraction of the VO_2_max reducing the rates of heat production and glycogen degradation [56]. This outcome can be supposed to lower the stress that pentathletes have to deal with during the many hours they train each day.

#### 4.3.2. Peak Oxygen Uptake (VO_2_peak)

The pentathletes of the present study reached peak oxygen uptake values (VO_2_peak) of 3540.1 ± 306.2 mL·min^−1^ or 48.8 ± 4.6 mL·kg^−1^·min^−1^. VO_2_max values of 4033 ± 655 mL·min^−1^ or 52.4 ± 248.8 ± 4.6 mL·kg^−1^·min^−1^ for elite swimmers, ranking in the 764 ± 44 FINA points, have been reported. Based on their different swimming skills and proficiency, lower values were expected for pentathletes relative to swimmers.

It must also be noted that we assessed VO_2_peak in a flume by a multistage incremental protocol up to exhaustion. For this reason, some physiological and psychological limits were expected because performance assessed using exercise simulators can differ from those executed in the field for their bioenergetic, biomechanical and mental characteristics [27,66]. It is possible that pentathletes struggled to keep their position in the swimming flume, focusing on trying not to be carried away by the water flow rather than to give their best effort [67]. Indeed, test swimming velocities were determined for the athlete to become exhausted within 8–12 min, as generally recommended for incremental exercise test in which VO_2_max is determined [54,56]. However, pentathletes were exhausted after 6:30 min only, at a mean velocity of 1.27 m·s^−1^, which is significantly faster than their swimming competition velocity. The noticeable short test duration seems to validate the difficulty for the pentathletes to swim at a fast speed and in the flume.

The flume ergometer swimming condition could not only account for the low VO_2_peak achieved but also for the lack of its correlation with the 200 m performance in the pool. Therefore, it appears that the VO_2_peak assessment in the flume does not constitute a meaningful protocol to ascertain pentathletes’ swimming ability and performance, and to ensure that the training load is producing effective, non-detrimental outcomes.

In elite endurance athletes, such as pentathletes, there is evidence that the VO_2_max is relatively insensitive to continued training and is a poor discriminator of performance capability. In these athletes, VO_2_max appears to reach high values early in the career and then to remain constant even though performance continues to improve [56]. Additionally, unlike other sports, VO_2_max and swimming performance has a mixed correlation. It could be possible that constraints in blood flow, oxygen transport and/or greater respiratory work during swimming could be responsible for important differences in VO_2_ from those that have been reported for upright exercise [68]. Nonetheless, the individual characteristics of the athlete will significantly influence internal load and stress placed on the body, which affects the athlete’s susceptibility to injury. It has been reported that the athlete’s aerobic fitness level will impact the internal workload they place on themselves [7]. For this reason, it seems important that multisport athletes complete a comprehensive assessment of the parameters of aerobic fitness for each discipline they compete in [56].

A limit to the interpretation of our results is the lack of kinematic parameters analysis. The relationship between biomechanical and physiological factors would have provided a more complete explanation for the presence or absence of correlation of the latter with race performance. Moreover, larger participant numbers and a greater variation in performance levels would have permitted clearer, more detailed results.

## 5. Conclusions

Our findings suggest that, among all the protocols analysed, CV can be the more predictive and discriminative of individual swimming performance in this group of pentathletes. The strong relationship between CV and swimming performance suggests that in pentathletes the distance–time relationship may be influenced by the technical level of the swimmer. The present study extended the CV utility to regularly monitor progress as well as to prescribe training loads specific to the athlete’s physiological and technical capacities.

## 6. Practical Implications

Swimming the 200 m freestyle underlines the critical involvement of both aerobic and anaerobic metabolism. The high-training volumes usually used in the training of competitive swimmers could be less advantageous compared to a high-intensity training of lower volume for this event. The most effective training load pattern for 200 m swimmers is characterized by a continuous high training load during the first six weeks, a low-, medium- and high-intensity training peak during the medium-term meso-cycle, and maintenance of low-to medium-intensity training during the short-term period. This implies the need to regularly adjust the training regimen throughout the season.

For elite athletes, the training modes that facilitate further progress are increasingly limited despite an increase in their underlying abilities. The possible loss of reactivity in the genetic and molecular response indicates the importance of a new and gradual overload that causes sufficient stimulus to induce new adaptations. Changes in training load of as little as 10% can make important differences to competition performance.

It seems plausible that relevant adaptations may be reached more economically if the training status of an elite athlete could be frequently and easily monitored. The individual, proper and precise assessment of the work rate that separates low-, high-, and severe-intensity domains is relevant to reveal the optimal training load for each athlete, in every period of the training season.

CV regular assessment could provide a strategy for coaches striving to deliver effective sessions despite limited pool time, such as in pentathlon. This measure can be extensively used by support staff to make decisions about training and rest prescription, depending on the athlete’s working capacity. Time and energy could be saved for other relevant training contents, such as the ability to sustain force and its application to the water. This could allow to maximize performance and reduce injuries, illnesses and overuse syndromes.

The strong correlation of CV with pentathletes’ swimming performance could also be of use to predict competitions’ outcomes and to determine a pentathlete’s talent for swimming. The assessment of CV could be suggested to be the most suitable to be applied to pentathletes’ swimming evaluation. Additionally, it is free and easy to administer, so that it can be incorporated into the longitudinal monitoring of training load and fatigue, which is crucial for injury prevention.

## Figures and Tables

**Table 1:** Statistical analysis procedure.

*Statistical Model*	*Purpose*	*Variables*	*Effect Size*
*Linear correlation*	Relation among variables	All variables	
*Paired Simple T-test*	VO_2_ differences	VO_2_ values	0.636
*Wilcoxon Signed Ranks Test*	Lactate values	Lactate accumulation	0.881
*Repeated Measure Anova*	VO_2_ and Energy cost	VO_2_peak and ECs	0.522
*Repeated Measure Anova*	Differences between times	Time values	0.998
*Repeated Measure Anova*	Velocities Differences	Velocity values	0.970

**Table 2:** Relationship (correlation coefficients) among the performance of the pentathlon competition swimming phase and the variables measured in the test sessions.

t200m Competition	100, 200, 300 m and critical velocity (CV)						
	t100m	t200m	t300 m	v100m	v200m	v300m	CV
	0.449	0.438	0.596 *	−0.448	−0.429	−0.624 *	−0.965 **
	Velocity corresponding to the 2 and 4 mM·L^−1^ of lactate accumulation (v2 and v4)						
	v200m		v200m at 90%			v2	v4
	−0.429		−0.450			−0.398	−0.304
	Peak oxygen uptake (VO_2_peak)						
	vPeak	Lapeak	VO_2_peak				
	−0.367	−0.211	0.001				
	Energy cost at submaximal velocity (EC)						
	ECrest	ECnet	∆La	ECLanet	TotECnet	TotECnet%	
	0.427	−0.257	0.15	0.15	−0.211	−0.068	

t100m, t200m, t330m: swimming times of the 100, 200 and 300 m tests; v100m, v200m, v330m: swimming velocities of the 100, 200 and 300 m tests; CV: critical velocity; v200m at 90%: submaximal velocity of 200 m swimming in test 2.1; v2, v4: velocities corresponding to the lactate accumulation of 2 mM·L^−1^ and 4 mM·L^−1^; vPeak, Lapeak, VO2peak: peak values of test 3. * *p* < 0.05; ** *p* < 0.01.

## Data Availability

the data analysed in the present study about the FINA points are publicly reported at https://www.fina.org/swimming/points; accessed on 15 June 2022.
